# Abstracts and Gleanings

**Published:** 1885-11-20

**Authors:** 


### ABSTRACTS AND GLEANINGS.

**Therapeutics of Jaborandi.**—M. Petithan, in a paper upon the therapeutic uses of jaborandi, claims its efficacy in the treatment of the following diseases :

1. In all catarrhal and rheumatic affections, subacute or chronic, whatever may be their localization.
2. In all dropsical conditions, without alteration in the kidney, when anæmia is not too marked.
3. In certain cases of pernicious intermittent fever, in which re-action is hastened without affecting the action of quinine.
4. In malignant febrile exanthemata, especially in scarlatina.
5. In diseases of the skin, characterized by dryness, psoriasis, etc.
6. In chronic syphilitic disease during the employment of mercury and the iodides.
7. In mercurial intoxication.
8. Probably in diphtheria.

The remedy, according to M. Petithan, should be used as follows:

1. It should be administered when the patient is fasting.
2. The dose should be according to the case, 60 grains being an average quantity.
3. The temperature of the infusion should vary according to the degree in which the sudorific effect is desired.
4. A local temperature of at least 66° F. should be constantly maintained.
5. The patient should not be permitted to leave his bed on any pretext unless well covered, in order to avoid variation of temperature and syncope, which may result therefrom.
6. Warm drinks should be administered, if the patient is thirsty, a condition very rare owing to the salivation produced by the drug.
7. The employment of a good quality of the drug, that of Continbo being the best.
8. To have at hand stimulants, such as ammonia, etc., to sustain the patient.
9. If necessary to administer an antidote, M. Petithan suggests the employment of belladonna.—*Medical News.*

**The Hand.**—If you meet with a bad injury of the hand, do not decide to amputate it too quickly, nor even a part thereof. Do not be in a hurry to sacrifice so much as a finger, nor even a part of a finge. The hand and every inch of the finger are too valuable to the mechanic to allow any of it to be removed in a moment of haste. Once lost, they cannot be replaced. Be conservative. It is far better to wait until the wounds are completely healed up, and then remove what may be in the way, or what may interfere with the action of the fingers. If sloughing and gangre should occur, wait until the line of demarcation is plainly discernable, it is time enough then to remove the already dead parts. Nature is a good teacher ; if we follow her leadings, she will guide us aright. —Prof. E. BORCK, in *New England Med. Monthly.*

**Antiseptics.**—It is now generally understood that antiseptic, means cleanliness. It does not matter what material you use; carbolic acid, bi-chloride of mercury, iodoform, or something else. If things are not perfectly clean, you will have no success. You have observed that I do not use any one article exclusively, but make use of them all, more or less, according to judgment and circumstances. I have discarded the carbolic acid spray during my capital operations, and, instead of carbolized water, I use the artificial serum. For general purposes I employ distilled water and salt.

No therapeutic agent must be abused. You have seen how carbolic acid is abused. Remember the cases which I have showed you. All of them came under my care after chills, fevers, and delirium had already set in. In these cases, the wounds looked angry and an erysipelatous inflammation extended some distance on all sides of the wounds. The relations became alarmed, the attending physicians wondered why all this came about in spite of the employment of carbolic acid, iron, and quinine. Now, why was all this? Simply for the reason that these doctors had prescribed carbolic acid—ordered the mothers to "put a few drops in water" and syringe the wounds well and often, to keep them clean—to prevent blood-poisoning, and thought of no other danger to the patient. The mothers think that if a little does good, a little more must do better, and so, by their careless treatment, they poisoned their patient with carbolic acid. In neither case had the physicians for a moment recognized the danger that threatened the patient.

How did I treat the patients? I ordered simple warm water applications to the wounds, and mag. sulph. 30 grains internally. In one case the latter prescription had to be repeated. In all, the symptoms passed off within forty-eight hours, and they were doing well under the dry permanrnt dressing.

Necer give such directions as "a drop or two," "or 10 or 20 drops in a cup of water." Always give positive directions. Specify accurately how many drops you wirh given. Do not trust the syringing of wounds to others; do it yourself, unless you have previously trained the nurse.

I often dress small wounds dry in their own blood. Here, remember, never put any water upon the wound. Do not touch the wound with the sponge; wash the parts clean all aronnd; cleanse the wound with dry absorbent cotton; bring the edges of the wound together, and you will have "union by first intention." Dress dry. It little matters what material you employ so long as it is clean.—*Ibid.*

**Treatment of Gonorrhœa in the Female.**—Dr. Hanks (in the Eastern Medical Journal) says: "It is most essential that you make frequent vaginal douches and copious—half gallon or more—of very warm water. In using these douches, corrosive sublimate, gr. I to Oi, chlorate of potash, ʒij to Oi, or carbolic acid, ʒss to Oi, will serve you well. Be sure that the patient is taught how to fill the vagina full with the fluid, by retaining the solution in the canal for a moment—ballooning it, as it were—by means of pressure over the vulva."

**Sajous on the Treatment of Hay Fever.**—The author groups the factors of hay fever thus: First, an external irritant; second, a predisposition on the part of the system to become influenced by this irritant; and third, a vulnerable or sensative area in which the system becomes influenced by the irritant.

This sensitive area is the portion of the nasal mucous membrane over which the branches of the ophthalmic nerves are distributed. From some cause these nerves become hyperæsthetic. By cauterizing the terminal ends of these nerves the connection between the excitable nerve centres and the external irritant is cut off, and so hay fever rendered impossible. There are three hypersensitive areas, called posterior, middle, and anterior, which, separately or together, are the principal seats of the hyperæsthesia in hay fever subjects.

The posterior area is most affected when reflex asthma is the most prominent symptom of the affection.

The anterior area is most affected when the head symptoms are prominent.

The middle area may be the starting point of all the symptoms combined.

In treatment, he suggests that all abnormal conditions of the nasal cavities, such as hypertrophies, polypi, exostoses, etc., must be removed before beginning the superficial cauterization at least six weeks before the expected attack of hay fever. Still, the treatment can be conducted during an attack of the fever. This method of treatment has been employed by many other workers in this field, with success in some cases.—*Detroit Lancet.*

**Foreign Bodies in the Ear.**—During the Spring you will, no doubt, have patients coming to you with bugs in their ears. The noise such creatures produce in the head is very distressing; the pain is very great. Do not attempt to remove the bug. Your chance to fail is ten to one. First kill the bug by pouring a few drops of chloroform into the ear, or, what is still better, have a bent glass tube ready, which should fit into the meatus, and run through a cork which fits into a bottle containing chloroform. Introduce the tube into the ear, hold it there for a minute, thus killing the bug first, then proceed to remove it.

Other foreign bodies, such as a grain of corn, a bean or pearl button, you can often remove without employing any anæsthesia, by doubling up three or four horse hairs into a small loop, pushing it between the foreign substance and the wall of the internal ear, then drawing the foreign body out. It will hook around the obstacle and can easily be pulled out.—*Ibid.*

**Mistletoe a Parturifacient.**—Dr. G. V. Hale (in the Texas Courier-Record of Medicine) says we have in mistletee a parturifacient equal to ergot. "In three recent cases of this description [parturition] the writer has exhibited a fluid extract of misletoe (*Phoradendron Flanescens*) prepared by Parke, Davis & Co., in doses of from 20 to 40 minims, repeated at intervals of twenty minutes, with the happiest results."

**Unna's New Bougie Treatment of Chronic Gonorrhœa.**—Every practitioner knows that a chronic gonnorrhœa, with a thickened mucous membrane, narrowing of the urethral lumen, *i. e.*, incipient stricture, cannot possibly be cured by injections. Their effect is neither sufficiently powerful nor localized, besides the stricture prevents the injected liquid from reaching all superior parts of the urethra, the frequent seat of a chronic stubborn gonorrhœa. It is Unna's merit to have first introduced the combined effect of mechanical dilatation and the simultaneous therapeutical impression of astringent or disinfecting drugs.

His treatment consists in the introduction of tin bougies, properly curved and coated with an ointment which dissolves only at the temperature of the body. His routine ointment consists of—

| | | |
|---|---|---|
| ℞. | Ol. Cacao | ℥ iii |
| | Ceræ flaræ | ʒ ss–i |
| | Arg. nitr | gr. xv |
| | Bals. Peruv | fʒ ss–fʒi |

He allows the bougie to remain in the urethra for ten to fifteen minutes. The ointment melts wholly, of course within two minutes, and fills the entire lumen of the urethra. Larger bougies are gradually introduced until complete removal of the stricture has been accomplished.

The stoppage of the gonorrhœal flux is rarely difficult under this treatment, even in cases of very old standing.

The so-called Swift's Vectores recently introduced to the profession in this country, are a decided improvement on Unna's Bougies, as they consist of soluble bodies without any metallic axis.—*Therapeutic Gazette.*

**Hypodermic Syringe.**—It is astonishing how few know or care how to keep this useful little instrument in good order and ready in at any moment for use. How often have I heard practitioners say, "I would have made a subcutaneous injection, but my syringe would not work."

If the leather upon the piston becomes dry—which it should never be allowed to do—soften it in cold water, *never* in warm. Leather should always be softened in cold water. (The same is true of youf sole leather splints.) After you have done this, soak it well in glycerine. It will then keep soft for a long time. If you put away your syringe, expecting not to use it for several weeks or months, put a few drops of glycerine into the syringe. Do the same with your aspirator. When you want it, it is ready, for the leather has been kept soft. After having used your syringe, clean with water, unscrew the needle canula, and, before putting the wire into the canula again, run it several times through a flame to evaporate all moisture. Always keep the point of your needle canulas sharp.—*Ibid.*

**Removal of Tape-worm.**—Mr. B., aged 45 years, a butcher by trade, had suffered for nine years from tape-worm. He had been treated by a number of physicians, but never had succeeded in getting rid of over three to four links at a time. He finally came to me for treatment, and I prepared a decoction from the bark of pomegranate according to Prof. Locke's formula: "Take half a pound of bark from the pomegranate root, add two and a half pints of boiling water, let the mixture stand in a warm place for two hours, then boil down to one pint; strain while hot through a fine wire strainer. To every six ounces of the decoction add one drachm of the fluid extract of jalap and five drops of the oil anise."

The patient had fasted from supper the night before, at which time he took a large cathartic. At 1 p. m. I gave five ounces of the preparation above named, and at 3 o'clock gave four ounces more. At 5 p. m. he passed an entire tænia solium, measuring thirty-five feet in length and one-half inch at its broadest part, gradually tapering down to almost a thread. Whether there was another worm left I have not been able to ascertain; if there is, I I shall follow the same treatment again. The patient is pursuing his usual avocation.—*Cincinnati Eclectic Medical Journal.*

**The Treatment of Typhoid Fever.**—(Da Costa in College and Clinical Record):

1. HYGIENIC.

Place the patient in a large. well-ventilated room, so that he may get plenty of fresh air. Allow but one person (nurse) with him. Keep friends away. Enjoin cleanliness. Keep patient washed twice daily with vinegar and water, or a solution of permanganate of potassium. Disinfect the dejections with carbolic acid or chloride of zinc, etc.

*Nourishment.*—There are times when the patient is weakest, as in the early morning; this is the case in all low fevers. Nourish him every two hours with beef or mutton broths, alternating with milk. Other broths, as chicken, etc. may be used. If the patient craves for more solid food, allow him at the mid-day meal a little arrow-root boiled in milk, or a soft-boiled egg. Excepting these, allow no form of solid aliment until convalescence is completely established, and even then be careful. *Be sure to feed the patient between* 4 a. m. *and* 5 a. m.; even wake him at this time to feed him. Allow a liberal supply of water, or toast water, ginger-syrup and water, or claret and water. It will keep the kidneys washed out.

2. MEDICAL TREATMENT.

Different plans have been instituted:

1. Quinine, which has been justly abandoned.

2. The mercurial plan—calomel, gr. v–x. per diem, at the first stage of fever—said to modify the intensity of the fever process. Not an effective plan.

3. Carbolic acid, gtt. i–ij. in mint water every two hours. This remedy is not to be relied upon.

4. Iodine treatment, as Lugol's solution, gtt. ij. four times a day. This promises something good in the way of treatment.

5. The plan used by Dr. Bartholow in the following combination:

℞. Acid. carbolic.................................f ʒ i

Tinct. iodinii.................................f ʒ ij

Dose, gtt. i–iij. every two or three hours. This is a good plan of treatment.

6. Dr. Da Costa's plan is by the use of mineral acids. Those that use this plan in Germany prefer sulphuric acid; in England, hydrochloric; in France, phosphoric; in America, nitro-hydrochloric acids. Of the last an ordinary prescription is gtt. xx. of the dilute acid in simple elixir. This will also control, to some extent, the diarrhœa.

Do nothing else if you can possibly get along without, but guard against complications, and treat them immediately as they arise.

The first prominent symptom to be noticed is the *diarrhœa.* If there are but three stools, unless they be unusually large, do nothing. If very profuse, give a little tinct. opii camphorata at night, or an opium suppository, gr. j. Should this fail, use:

℞. Bismuthi subnitrat.................................gr. x–xx

Opii.................................gr. ss–j

Every three hours. If this fails, try carbolic acid, gtt. j., with morphinæ sulph., every three hours. Often cupri sulph., gr. 1-12th, with opium, gr. 1-3d, is very effective.

*For the Tympany*, solid applications, or injections of vinegar, f ℥ j–ij. to water Oj. Internally administer turpentine, gtt. vij., in emulsion, with morphine, gr. 1-48th. Often strychnine is useful, but secondary to the above.

*Thoracic Symptoms.*—The pulmonary congestion occasions cough; the patient's position must therefore be changed frequently. If the patient is not too feeble, use dry cups. The internal use of turpentine is of avail when marked fever is associated with the congestion. Do not give expectorants. If there is a large accumulation of mucus, use aromatic spirits of ammonia.

*Sustain the circulation* by quinine in tonic doses, gr. vi–x., in the twenty-four hours, but alcohol is the best, in small doses, to keep up the heart's action. In the early morning increase the dose. Under stimulus the pulse of 150 should come down to 120 or 110. The first sound of the heart is the key to the amount required. From 4 to 10 ounces may be necessary. For nervous symptoms, as headache, delirium, etc., give opium with camphor or belladonna. Chloral is the most useful, but do not give it when the heart is weak.

*For high fever*, cold water is excellent. Put the patient in a bath until the temperature geis to 62°F. The tendency to intestinal hemorrhage is greater in this treatment than by quinine, which is next in importance, and should be given in doses of ℈j–ʒss. in the day.

*For intestinal hemorrhage*, ergotine, gr. i.j–vij., hypodermically, or f ʒ j. fluid extract of ergot may be given every hour or two.

Sulphuric acid is also useful. Opium, to keep the bowels at rest, is indispensable. Cut down milk and stimulus now.

*Spreading tenderness* (peritonitis). Tinct. opii deodorat., gtt. x. every hour, and gr. j. opium suppository at the same time. The suppository must not be repeated for four hours.

Should the patient have *parotiditis*, ice is the best treatment; also tinct. ferri chloridi, to enrich the blood.

**Poisoning by the Oil of Sassafras.**—In the Trans. Maryland State Medical Society, 1884, p. 283, Dr. Chas. G. Hill reports the following observation: An office boy in closing his employer's office saw a bottle of sassafras. Remembering that he had heard that this would remove an eruption on his face, he turned up the bottle and took two large swallows. In a few moments he began to feel stiff. He still endeavored to raise the window to close the shutters. His strength now failed, and both he and the window dropped. The noise made by these falls attracted the attention of a policeman. He found the boy lying unconscious on the floor alone. Being taken to the station house, he was dragged about by the officers on the supposition that he was poisoned with opium. He spoke occasionally, but begged to be allowed to sleep. He was greatly relaxed, skin covered with perspiration, countenance pallid, pulse rapid but weak and thready. His pupils were normal, and there was a strong odor of sassafras in his breath. On being seen by a doctor, an emetic was given, followed by a copious emesis redolent with the odor of sassafras, with drops of the undissolved oil floating in the liquid. This was followed by free draughts of warm water till only a faint odor of sassafras was discoverable. After this he was soon relieved and restored to consciousness. He felt no discomfort, except the sensation of weakness and exhaustion.—*Detroit Lancet.*

**For Stutterers.**—A gentleman who stammered, from childhood almost up to manhood, gives a very simple remedy for his misfortune: "Go into a room where you will be quiet and alone, get some book that will interest but not excite you, and sit down and read two hours aloud to yourself, keeping your teeth together. Do the same thing every two or three days, or once a week, if very tiresome, always taking care to read slowly and distinctly, moving the lips, but not the teeth. Then, when conversing with others, try to speak as slowly and distinctly as possible, and make up your mind that you will not stammer. Well, I tried this remedy, not having much faith in it, I must confess, but willing to do almost anything to cure myself of such an annoying difficulty. I read for two hours aloud, with my teeth together. The first result was to make my tongue and jaws ache, that is while I was reading, and the next to make me feel as if something had loosened my talking apparatus, for I could speak with less difficulty immediately. The change was so great that everyone who knew me remarked it. I repeated the remedy every five or six days for a month, and then at longer intervals until cured.—*Phys. and Surg. Invest.*

**Gelsemium Habit.**—Dr. H. C. Caldwell, (Medical and Surgical Herald,) says:

The subject of this communication was twenty-four at the time the writer first met him. He was robust, had lived a life well divided between work on the farm, study, and rational recreations. Mentally he was of that type we style *well balanced*. He was, therefore, not such a person as we would expect to see become the victim of a habit.

He contracted chills, rheumatism supervened, and he refused the frequent offers made him of chloral hydrate and morphia. I may here state that his father had been addicted to the opium habit, and the son grew up with a horror of the very name of opium. In an attack of more than usual severity he took a large dose of fluid extract of gelseminum. Relief followed. The next day a repetition of the paroxysm called for a repetition of the dose. As with all quieting agents, the dose must be augmented, and during the year this increase was not very great.

One hot night, in great agony, the sufferer took a very large dose and lay stupid until noon the next day. The experiences he had were, as he said, "woderfully pleasant."

Now the habit became fixed. The victim grew to using as much as a fluid ounce of the extract at one dose! What would once have produced death, was now only a gentle palliative. Still the dose must be increased.

He became pale, emaciated, listless, and, at times, strangely uneasy. He became the prey of strange terrors, and was subjected to some hallucinations of the physical senses.

Looking fixedly at any distant object, he could discern all the colors of the spectrum; then darkness followed, and then a number of faint rays of light would precede the complete return of vision.

His hearing became singularly acute. He was apparently regardless of what was passing; still he could detect whispers, he told me—whispers uttered many yards away.

Nothing could induce my poor friend to give up his darling drug. Seeing how the matter distressed his friends, he went away. During his stay of a year in Canada he increased the dose daily. He returned far more feeble, and, at times, seemed positively idiotic.

He fancied ghosts were around him; he could hear the shrill whispers of leering demons, and, in his better moments, saw the starry wings of angels hovering around his bed.

After a year more of this strange habit, he sank into a coædition of hopeless idiocy, and died in the stupor induced by his idolized drug.

The relatives of the unfortunate man never took care to prevent his obtaining the drug, but this neglect is seen in regard to the victims of the opium habit every day. It is to be hoped that in an advanced state of civilization—the true and better sort of civilization—such persons will be taken away from the care of neglectful and ignorant kinsmen, and placed in public institutions where judicious medical treatment and the proper moral suasion shall be exerted as to redeem many a poor creature from those hideous vices "that wreck the body and debase the mind."

**Progress in Medicine.**—Dr. Arch. Dixon, (Medical and Surgical Reporter) in a parper read before the Mississippi Valley Medical Society, Sept. 9, 1885: A review of the history of medicine during the past year will bring to our attention much of interest. Solid advances have been made in methods of treatment, remedies of value have been added and the usefulness of older ones confirmed. The introduction of cocaine as a local anæsthetic, had nothing more been done, would mark it as a year ever to be remembered by our profession. While other lands have been devastated with epidemics, ours has been exceptionally healthy, excepting a local epidemic of typhoid fever at Plymouth, Pa. Yellow fever and cholera have given us a wide berth. To-day the great question is, Is cholera a preventable disease, and if so, can it be averted from our shores? There is an old saying, that history repeats itself, and it is eminently true of the history of cholera. Beginning in India, where it is endemic, especially true of Bengal, where every third, sixth, ninth and twelfth years the great pilgrimages to Juggernaut and affiliated shrines assemble, it spreads slowly westward, following the routes of the returning pilgrims and in the course of traffic by land and sea, until it reaches Europe, where it prevails a year or two before it is brought to this country. The influences of these pilgrimages on the epidemics of cholera can be traced from 1781 to the present epidemic in Spain and France. Unless measures are taken to prevent it, we shall have a repetition of those fearful visitations which beginning at Quebec in 1832, extended to the Gulf, spreading horror and desolation in its track. There is no doubt that each and every visitation has been the result of importation.

Now, the point to be determined is, is the germ origin of the disease the true one? Is the germ always present in cholera; is it ever present elsewhere? Koch maintains there is a definite organism associated with cholera, and this opinion is supported by many of the best men of our profession, both in this country and in Europe.

After discussing the bacillus question in all its phases, he thought that it followed that the poison of cholera must be a micro-organism, for nothing but a living thing can reproduce itself, and I consider that Koch's position so far has remained unassailable. If then the germ theory of cholera be true and it be a contagious and portable disease, then as a matter of course it must be admitted that it is a preventable disease, and the point at once arises how best to destroy these germs? The answer is by disinfection and the proper use of germicides, with a sufficient quarantine to guarantee detention until the process is complete. Detention without disinfection is not effective. With the efficacy of germicides, quarantine should become what it is intended to be, a delusion, and not a snare. There is no better plan presented than that of Joseph Holt, M.D., president of the Louisiana State Board of Health, which the doctor presented in his paper. Perhaps in New Orleans, whose people have been so often scourged, these precautions of Dr. Holt may be carried out, but not elsewhere.

Dr. Rauch, (North American Review, August, 1885,) Secretary

State Board of Health of Illinois, says that if no vessel were allowed to leave a port infected with cholera, or carrying persons or things from an infected region without being first made secure against the possibility of carrying the disease, it would of itself render unnecessary all other means of combating cholera so far as we are concerned. But this has never been accomplished.

During the existence of Asiatic cholera in Europe this country is only safe when a system of sanitary supervision is practiced over the immigrant and his effects. The instructions of our best sanitarians if carefully heeded will demonstrate that cholera can be kept from our country.

The Italian Society of Royal Hygiene have arrived at conclusions which, owing to their experience with the cholera during the past year, are worthy of receiving great consideration. These conclusions the doctor quoted *in extenso.*

**Hydronapthol.**—Dr. Levis, in Medical and Surgical Journal, says: Hydronapthol, the new antiseptic recently introduced into the surgical world by Dr. George R. Fowler, of Brooklyn, bids fair to supersede most of the antiseptics now in common use, as its claims are undoubtedly stronger than those of any one agent at our command.

It is antiseptic in the truest sense of the word—*it prevents putrefaction.* Its action is chiefly inhibitory, and excepting corrosive sublimate, it is most powerful in this particular.

To review what Dr. Fowler has brought forward in its favor—

1. It is non-irritant, non-poisonous, and non-corrosive.

2. Though only solable in water to the extent of one part of hydronaphthol to one thousand parts of water, in this proportion it is antiseptic.

3. It is inodorous, hence, cannot disguise the odor of putrefaction.

It is not decomposed nor rendered inert by the products of purefactive decomposition—such as sulphuretted hydrogen, ammonia, etc. It is not volatile at the ordinary temperature of the atmosphere, hence is more stable than carbolic acid, than which it is fifteen times more efficient. It is positively harmless alike to tissues and fabrics.

Being non-corrosive, it will not injure the polished surfaces and keen edges of cutting instruments, and is in this respect more desirable than corrosive sublimate.

A saturated solution, as above stated, is about the strength of 1 to 1000, and in this proportion, perfectly preserves for an indefinite time, animal tissues and fluids ; yet, upon living tissues this solution has no other perceptible effect than the formation of a slight albuminous film—this being rather an advantage than otherwise, as it secures against infective germs floating in the atmosphere.

It is easily powdered, and in this state triturated with carbonate of magnesia or oxide of zinc, etc., etc., in the proportion of two parts of hydronaphthol to 100 parts of 1 of the above-named substances (oxide of zinc probably the best) may be dusted over the wounds, along the lines of incisions, and over the mouths of drainage-tubes—in this latter application, it presents an advantage over iodoform, now so commonly used, in that it does not dry up the serum escaping from the wound cavity, and thus block up the exit extremity of the tube.

Its ten per cent. (alcoholic) solution perfectly sterilizes silk, and hardens, sterilizes and preserves cat-gut.

Though as an antiseptic it proved active in arresting the development of bacteria in the proportion of 1-1000 parts, it did not stand the test of a germicide in a solution five times above saturation.

Here, as elsewhere, holds good the old proverb: "An ounce of prevention is worth pounds of cure." If we prevent the formation of sepsis in a given case, we will obviate the necessity for a germicide or disinfectant.

This week, for the first time, we relied upon hydronaphthol as the sole antiseptic used in a case of compound fracture of the leg. When the patient was admitted to the hospital, he had a temperature of 100°F.; now, at the end of the fourth day, it is normal, and the wound doing well in every respect.

As a true germicide, for use where sceptic conditions already exist, the bichloride of mercury is the most efficient agent; for simple antisepsis, or inhibitory or preventive action, hydronaphthol appears to be preferable for general use, and may well displace carbolic acid.

**To Prevent the Secretion of Milk in Still-born Cases.**—A writer in The Investigator says: Pressure. promptly and thoroughly applied, at latest within a few hours after labor, before the determination of blood has set in in full force, will, by preventing the glands from filling, force the blood onward into the general circulation and meet the requirements of the case to a nicety. But how are we to apply this pressure? Strapping with strips of adhesive plaster is usually recommended; but who has ever succeeded in strapping the breasts artistically, efficiently, and satisfactorily! Take two pieces of surgeons' plaster, light and wieldy, each nine inches square—one for each breast—and lay them on so as to see where to cut out circular pieces, which, when removed, will leave holes for the nipple to pass through. Fit the plasters to the breasts before applying them; snip or gore them in the sides to take out wrinkles; and then put them on tight and snug from the bottom, with strong traction upwards. A piece of adhesive plaster, nine inches square, if applied immediately after labor, is generally large enough to extend beyond the limits of the gland, and has as *points d'appui*, the ribs above and below and the sternum in the mid-line. The gland, in this way, is squeezed equably against the chest, and bound there; expansion is prevented; the sense of weight never experienced; the blood supply forced into other channels, and any milk secreted beyond the present capacity of the breast is forced along the natural channels and discharged at the nipple. The patient's attention, too, is directed away from the breasts, since they do not cause her any inconvenience, and one more fertile cause of worry is removed from her mind. Milk may continue to be secreted in small quantities for a couple of weeks, after which the glands become inactive; but this secretion is not wholly objectionable, since it may accelerate uterine involution.

The same method of applying pressure so as to dry up the breasts, may be successfully employed at weaning time; as also in the case where the child has nursed for some time and died when the milk flow was well established; and when, through a mammillary deformity, we are forced to dry up one breast without interfering with the other. In this last case, of couise, we would only strap up the blind breast; in the two first instances we might advise, as an additional aid, an abstention as complete as possible from fluids and the exhibition of a saline cathartic. In all three cases, however, the precaution should be taken to completely empty the breasts or breast before applying the plaster, so that accumulation of milk will be impossible, once the plaster has been put on properly.

**New Method of Reducing Dislocations of the Hip.**—Dr. S. J. Allen, M.D.: An anæsthetic having been administered to the extent of producing complete muscular relaxation, the surgeon stands over the recumbent patient, whose leg he flexes upon the thigh, and the thigh raises to a right angle with the body, bringing the foot between the surgeon's legs, so that its dorsum rests against his nates. If then the surgeon, passing his right arm beneath the flexed knee, lifts the hips of the patient well from the bed or floor, and holds them thus suspended for a very short time, the head of the femur will quickly be drawn into its socket. The weight of the hips and opposite leg rotates the body outwards, producing just sufficient abduction and extension to quietly draw the head of the femur through the slit in the capsular ligament, and direct it into the acetabulum.—*Annals of Surgery.*

**Tonsillitis.**—M. Gine, of France, claimed that by the use of bicarbonate of soda he was able to cut short an attack of tonsillitis twenty-four hours after the application of the powdered salt to the tonsil. Such rapid cure has, however, never occurred in Mr. Kendall's practice, but he has had very good results in thirty-six and forty-eight hours, although in elderly people the attack has lasted five or six days. The drug is well worthy the attention of the profession on account of the absence of harmful properties, and is, without doubt, also useful in cases of an inflammatory sort, either due to the influences of cold or to an exhausted nervous system.—*Ex.*

**Potassium Bromide in Ovarian Menorrhagia.**—Dr. Alfred Meadows (Inaugural Address, British Gyneological Society), said that every one would concede that potassium bromide exercised a most powerful influence upon the ovaries. No drug, in his experience, equaled it in controlling ovarian menorrhagia; it not only limited the flow in these particular cases, but seemed to exercise a distinct and controlling influence upon ovulation itself, decreasing its frequency.—*Obstetric Gazette.*

**The Last Episode of the Ferran Inoculations for Cholera.**—Under this heading the Union Medicale, October 6, publishes an extract from a communication in the Spanish paper, the Diario, of Tarragona, concerning an alarming consequence that has followed the Ferran inoculations at a small maritime locality called Cambais, situated between Tarragona and Tortosa. No particnlars of the number of inoculations that have been effected are given, but it is asserted that nine of the persons who had undergone inoculation, had subsequently been obliged to undergo amputation of one or both arms, in order that the gargrene which ensued might be arrested. At the time the communication was made there were two other persons about to undergo the same operation. A complete panic has arisen in the neighborhood, especially among those who have undergone inoculation.—*Medical and Surgical Reporter*.

**Veratrum as a Diaphoretic.**—A writer in the Georgia Eclectic Medical Journal says of veratrum :

" Aside from the pulse, another indication for veratrum vir. is a dry tongue, and mucous membranes generally show lack of secretion. These are sufficient indications. Now for the dose, say for adults :

℞. Tr. ver. vir. (Norwood) ...............gtts. vij to xij,
Aq. two-thirds of a goblet. M.

" Sig : One teaspoonful every half to one hour until it produces a diaphoretic effect, both in the external and internal skins, *which it will do*, and will thus relieve the blood.

" For children, according to age, etc., from gtts i to iij ; aq. say two-thirds of a goblet. M. Sig : Dose as above.

" If the determination is mostly to the lungs, add to the mixture a little sugar to assist as an expectorant."

**The Microbe of Mumps.**—M. Ollivier, it is said, has succeeded in discovering definite forms of microcci in both the saliva and the urine of subjects suffering from mumps. These microcci exist either singly, or united in pairs, or in the form of a zooglœa mass ; and in addition to them the fluids examined contained the bacteria usually found in such excretions.—*Ib.*

**The Operation of Turning.**—When you cannot find the feet of the child, in the operation of turning, reach for the fundus of the uterus, and when there open the hand widely and withdraw it slightly ; the feet will then come into the hand of the operator. This operation is often facilitated by the knee-chest position.—*Medical World.*

**Vomiting in Pregnancy.**—It is stated in the Medical and Surgical Reporter that carbolic acid, or Apollinaris water, injected into the rectum relieves the vomiting in pregnancy. Apollinaris water may be the best to use in these cases.

**An Antiseptic Ointment.**—Dr. M. B. Ward, of Topeka, Kans., sends the formula of an antiseptic ointment which he found very useful while in charge of the hospital department of the Mexican Central Railway. It consists of—

℞. Iodoform................................ʒ j,

Subnitrate of bismuth.......................ʒ vij,

Vaseline....................................℥ ij.

This formula was not strictly adhered to in all cases, but was varied according to individual indications. Another formula, suggested by Dr. J. W. Thayer, was—

℞. Iodoform................................ʒ j,

Boracic acid................................ʒ ij,

Subnitrate of bismuth.......................ʒ iv,

Vaseline....................................ʒ ij.

The ointment was spread upon a sheet of absorbent cotton sufficiently large to cover the wound and a considerable extent of surface around it. "The dressing seemed to be the best suited to the after-treatment of amputation wounds. It could be removed when necessary with the greatest facility, as it never adhered to the wounded surface. I usually allowed the first dressing to remain undisturbed for from four to six days. There never was present, even in the warm climate of Mexico, the least disagreeable odor, so common when carbolic dressings are used." This dressing was used in over four hundred cases of injuries of various kinds, including ten gunshot wounds and numerous amputations and other capital operations, and of this number there were but two deaths.—*New York Medical Record.*

**Protective for Exposed Dental Nerves.**—Ten parts of collodion to fifteen parts of creasote (Medical World) form a firm gelatinous mass of great efficacy, as a stopping and protective to exposed nerve-substance in dental cavities.

**For Urethral Discharges.**—Dr. Ferrier's snuff, dissolved in water, makes an excellent injection for urethral discharges, as gleet, gonorrhœa, etc. It consists of six drachms of subnitrate of bismuth, two drachms of powdered gum acacia, and two grains of morphia. This quantity may be dissolved in six ounces of water, to be used with a small syringe.—*Medical World.*

**To Keep Mosquitoes Away.**—The effectiveness of the following mixture for keeping off mosquitoes is vouched for by The Angler : Three parts olive oil ; two parts oil of pennyroyal ; one part glycerin ; one part ammonia. To be well skaken before applying to the face and hands. Avoid getting the mixture in the eyes.—*Northwestern Lancet.*

**A Cheap and Efficient Disinfectant.**—A drachm of corrosive sublimate to a gallon of water constitutes a cheap and efficient disinfectant for general purposes.—*Medical World.*

